# Humanin (HN) and glucose transporter 8 (GLUT8) in pregnancies complicated by intrauterine growth restriction

**DOI:** 10.1371/journal.pone.0193583

**Published:** 2018-03-28

**Authors:** Carla Janzen, Margarida Y. Y. Lei, Il Seok D. Jeong, Amit Ganguly, Peggy Sullivan, Vladislava Paharkova, Gina Capodanno, Hiromi Nakamura, Alix Perry, Bo-Chul Shin, Kuk-Wha Lee, Sherin U. Devaskar

**Affiliations:** 1 Department of Obstetrics and Gynecology, Division of Maternal-Fetal Medicine, David Geffen School of Medicine at UCLA, Los Angeles, California, United States of America; 2 Department of Pediatrics, Division of Endocrinology, David Geffen School of Medicine at UCLA, Los Angeles, California, United States of America; 3 Neonatal Research Center of the UCLA Children’s Discovery and Innovation Institute, David Geffen School of Medicine at UCLA, Los Angeles, California, United States of America; 4 Department of Pediatrics, Division of Neonatology, David Geffen School of Medicine at UCLA, Los Angeles, California, United States of America; 5 Department of Pathology and Laboratory Medicine, David Geffen School of Medicine at UCLA, Los Angeles, California, United States of America; Virgen Macarena University Hospital, School of Medicine, University of Seville, SPAIN

## Abstract

**Background:**

Intrauterine growth restriction (IUGR) results from a lack of nutrients transferred to the developing fetus, particularly oxygen and glucose. Increased expression of the cytoprotective mitochondrial peptide, humanin (HN), and the glucose transporter 8, GLUT8, has been reported under conditions of hypoxic stress. However, the presence and cellular localization of HN and GLUT8 in IUGR-related placental pathology remain unexplored. Thus, we undertook this study to investigate placental expression of HN and GLUT8 in IUGR-affected versus normal pregnancies.

**Results:**

We found 1) increased HN expression in human IUGR-affected pregnancies on the maternal aspect of the placenta (extravillous trophoblastic (EVT) cytoplasm) compared to control (i.e. appropriate for gestational age) pregnancies, and a concomitant increase in GLUT8 expression in the same compartment, 2) HN and GLUT8 showed a protein-protein interaction by co-immunoprecipitation, 3) elevated HN and GLUT8 levels *in vitro* under simulated hypoxia in human EVT cells, HTR8/SVneo, and 4) increased HN expression but attenuated GLUT8 expression *in vitro* under serum deprivation in HTR8/SVneo cells.

**Conclusions:**

There was elevated HN expression with cytoplasmic localization to EVTs on the maternal aspect of the human placenta affected by IUGR, also associated with increased GLUT8 expression. We found that while hypoxia increased both HN and GLUT8, serum deprivation increased HN expression alone. Also, a protein-protein interaction between HN and GLUT8 suggests that their interaction may fulfill a biologic role that requires interdependency. Future investigations delineating molecular interactions between these proteins are required to fully uncover their role in IUGR-affected pregnancies.

## Introduction

Intrauterine growth restriction (IUGR) results from the inability of the fetus to reach its full growth potential due to conditions that impede normal achievement of expected weight for any gestational age. IUGR is traditionally defined as an estimated fetal weight (EFW) less than 10^th^ percentile for gestational age and sex, however, this definition includes normally grown but “constitutionally” small fetuses. Distinguishing between normal and pathological growth is essential in clinical practice, as IUGR is the second leading cause of perinatal morbidity and mortality worldwide, and has important implications for adverse endocrine function and metabolism throughout life [1]. The pathogenesis of IUGR is poorly understood, but is thought to involve placental insufficiency with concomitant disruption of nutrient supply, the redox balance, and energy metabolism [2, 3].

Although hypoxia regulates the physiological development of human embryos, persistent hypoxia presents a significant stress to the growing fetus [4]. Based on studies demonstrating its cytoprotective role in diseases related to persistent hypoxia, we hypothesized that humanin peptide (HN) may be important in mediating such stress in IUGR [5–8]. Originally described by several groups [9–12], HN is a small, endogenous peptide that is putatively encoded within the open reading frame of the 16S rRNA of the mitochondrial genome. Since its discovery, HN has shown to provide cytoprotection against various cell stressors in several organ systems[12]. We have demonstrated that treating mitochondria, a major source of reactive oxygen species, with HN reduced H_2_O_2_ production over 50%, supporting a direct role for HN in reduction of mitochondrial oxidative stress[8]. Other studies have also shown that addition of exogenous HN under a number of oxidative stress-associated conditions to be cytoprotective[5–8].

Multiple studies of humanin expression in skeletal muscle suggest that humanin expression may be up regulated in conditions of oxidative stress as a physiological response to cope with defects in mitochondrial energy production[13]. In addition to its cytoprotective/anti-apoptotic role, HN has been linked to glucose homeostasis by improving glucose-stimulated insulin secretion and peripheral insulin signaling [14]. Recently, serum humanin concentrations were found to be increased in women affected by preeclampsia [15]. Given the increased levels of HN [16] and GLUT8 [17] under conditions of oxidative stress observed in non-placental systems, and the intracellular localization of both, we hypothesized that there may be a similar expression profile of HN and GLUT8 in the placenta, and that expression of these may be altered in pregnancy affected by IUGR.

Glucose is a critical nutrient for the fetus, relying on transport at the maternal-fetal interface. Perturbations in this transport system also contribute towards the development of IUGR. There are 14 known isoforms of the membrane-spanning glucose transporter (GLUT) family, which are responsible for facilitated diffusion of glucose across the lipid bilayers of cell membranes [18]. Several members of the GLUT family, including GLUT1 [19, 20], GLUT3 [21–23], GLUT4 [24], and GLUT8 [25] are expressed by the human placental trophoblasts during pregnancy. In a previous study, we found chronic hypoxia plays a critical role in altered placental GLUTs [26]. While no changes in GLUT1 and GLUT4 were seen, the expression of the plasma membrane associated GLUT3 was increased in trophoblasts on the maternal face of the term placenta in IUGR compared to normal, appropriate for gestational age term placenta. This elevation was associated with increased nuclear concentration of HIF-1α, suggesting that hypoxia may play a role in up-regulating GLUT3. However, GLUT8, a related isoform also expressed in the placenta with an intracellular localization [27], has not been examined so far in the human IUGR term placenta.

Here, we describe HN and GLUT8 for the first time in IUGR-affected placentas and the expression of these proteins in placentas from IUGR-affected versus normal pregnancies.

## Materials and methods

### Placental clinical characteristics and tissue collection

Our study protocol was approved by The University of California, Los Angeles (UCLA) Institutional Review Board (IRB). For participation in this study, subjects provided consents for use of study samples for research and use of their medical records. After informed consent was obtained, placentas were collected at the time of delivery at the UCLA Labor and Delivery Unit. Twenty-two placentas were collected from normal term births, and twenty-two placentas were from term IUGR births [28] (birth weight less than the 10^th^ percentile) that demonstrated a trajectory of fetal growth deceleration *in utero*, diagnosed by prenatal ultrasound [29]. To meet criteria for eligibility in this study, subjects whose pregnancies were complicated by idiopathic, uncomplicated IUGR were included (we did not include cases that had additional clinical complications; thus these subjects did not have preeclampsia, diabetes, or other significant co-morbidities). The maternal age, gestational age at delivery, placental weight, birth weight, baby sex, and delivery modes are in Table 1.

**Table 1:** Clinical characteristics of control and affected groups. Values are ±SEM. Statistical significance was assigned at p<0.05 using Student’s *t*-test.

	Normal Pregnancy	IUGR-Affected Pregnancy	P-Value
n =	22	22	
Maternal Age at Delivery (Years)	32.227 ± 1.369	29.727 ± 1.043	0.085
Gestational Age at Delivery (Weeks)	39.104 ± 0.223	38.054 ± 0.387	0.026*
Placental Weight (Grams)	646.252 ± 39.25	414.601 ± 24.991	<0.001*
Birth Weight (Grams)	3361.182 ± 62.084	2450.895 ± 66.019	<0.001*
Baby Sex	9 females, 13 males	9 females, 13 males	
Delivery Mode	16 c/s, 6 NSVD	7 c/s, 15 NSVD	

* = p<0.05.

IUGR-affected pregnancy parameters were statistically different from their corresponding normal groups.

Detailed characteristics of the IUGR patients are in Table 2. Fifteen out of twenty-two IUGR babies were asymmetrically grown (Table 2). Asymmetric IUGR was defined by birth weight less than length and/or head circumference percentiles when the weight percentile was two percentile categories below length and/or head circumference [30].

**Table 2:** Clinical characteristics of IUGR cases.

	Birth weight, gm	Percentile(%)	Gestational age (wk)	Length (cm)	Percentile (%)	Head circumference (cm)	Percentile (%)	Growth profile	Adverse outcome due to IUGR	
									Antenatal	Postnatal
1	2240	<3	39+2	46	3	33.5	20	asymmetrical	1. AC (<3%) 2. Abnormal Dopplers	-NICU admission
2	1865	3	35+4	44.5	20	29.5	10	asymmetrical	1. AC (<5%) 2. Abnormal Dopplers 3. Oligohydramnios	-NICU admission
3	2240	3	35+5	46	10	31	7	asymmetrical	-Fetal growth deceleration	-NICU admission
4	2190	3	37+6	47.3	15	30.5	3	asymmetrical	-C/S for fetal distress	-NICU admission
5	2300	3	38+1	45.5	3	33	30	asymmetrical	-Fetal growth deceleration	
6	2495	7	38+1	47	10	30.5	3	asymmetrical	-Fetal growth deceleration	-NICU admission
7	2645	7	39	49	30	32.5	10	asymmetrical	1. Fetal growth deceleration 2. Abnormal Dopplers	
8	2552	7	38+2	50	50	33	20	asymmetrical	-Fetal growth deceleration	-NICU admission
9	2475	8	37+3	48.3	45	33	30	asymmetrical	1. Fetal growth deceleration 2. Abnormal Dopplers	
10	2560	8	38	51.4	80	33.5	40	asymmetrical	1. Fetal growth deceleration 2. Abnormal Dopplers	-NICU admission
11	2890	8	40+3	50.8	40	33.7	10	asymmetrical	1. Fetal growth deceleration 2. Abnormal Dopplers	-NICU admission
12	2535	10	37+4	48.9	50	32.4	30	asymmetrical	-Fetal growth deceleration	
13	2980	10	40+1	50.8	40	30.5	<3	asymmetrical	1. AC (<3%) 2. Fetal growth deceleration 3. Abnormal Dopplers	-NICU admission
14	2675	10	37+4	49.5	50	33.7	50	asymmetrical	-Fetal growth deceleration	1. NICU admission 2. Fetal CHD
15	1920	10	34+2	40.5	3	31	30	asymmetrical	-Fetal growth deceleration	-NICU admission
16	2580	2	40+3	46.5	3	31	<3	symmetrical	-C/S for fetal distress	-NICU admission
17	2840	3	41+2	48.4	7	33.2	7	symmetrical	-Fetal growth deceleration	-NICU admission
18	2690	7	39+3	48.3	10	31.8	3	symmetrical		-NICU admission
19	2460	7	37+6	43.2	<3	30.5	3	symmetrical	1. Fetal growth deceleration 2. Abnormal Dopplers	-NICU admission
20	2608	7	39	44.5	<3	33	10	symmetrical	-Fetal growth deceleration	
21	1860	7	35	44	10	29.9	9	symmetrical	1. AC (<3%) 2. Abnormal Dopplers 3. Oligohydramnios	-NICU admission
22	2320	9	36+5	45.3	10	32.3	25	symmetrical	1. Fetal growth deceleration 2. Abnormal Dopplers	-NICU admission

A cross-section of placental tissue was obtained at the insertion of the umbilical cord. The amnion membrane was peeled off the cross-sectional placental slice and the tissue washed in ice-cold 1x phosphate-buffered saline (PBS) to remove maternal blood. Fetal placental samples were obtained from the area close to the amnion. Maternal samples were collected from the basal plate after the decidual layer was removed by sharp dissection as previously described [26]. One set of samples was snap-frozen in liquid nitrogen and stored at -80°C, and another set of samples was formalin-fixed for immunohistochemical analysis.

### *In vitro* trophoblast cell studies

#### Trophoblast cell culture and treatment

We obtained the human extravillous trophoblast (EVT) cell line, HTR8/SVneo, developed by Dr. Charles Graham (Queens University, Kingston, ON, Canada) [31] as a gift provided by Dr. Ravi Javeri at Duke University Medical Center. Gibco reagents (Life Technologies, NY) were used for cell culture studies. Cells were cultured in a gas mixture of 5% CO_2_-95% air at 37°C in RPMI 1640 medium with L-glutamine and 25 mM HEPES and 5% penicillin-streptomycin supplemented with 10% fetal bovine serum (FBS), and were passaged using 0.25% trypsin-EDTA to maintain 70–80% cellular confluence.

After HTR8SVneo cells were plated in complete media for 24 hours, 2 experiments were performed to simulate conditions of a) hypoxia and b) growth factor deprivation. For hypoxia, HTR8SVneo cells were treated with cobalt chloride (CoCl_2_), a chemical mimetic of hypoxia (Sigma-Aldrich, MO). We have chosen to administer cobalt chloride at concentrations of 250 μM and 500 μM, based on prior studies that tested COCl_2_-induced hypoxia in HTR8/SVneo cells [32]. For growth factor deprivation, we incubated cells with serum-free media (FBS is omitted). After 24 hours, cells were lysed in cold RIPA lysis buffer with a protease and phosphatase inhibitor cocktail (Thermo Scientific/Pierce, IL), sonicated, and centrifuged at 4°C. The cell lysates were analyzed by western blot to assess HN and GLUT8 expression.

#### RNA extraction and quantitative RT-PCR

Total RNA was extracted from human placental samples using Direct-zol RNA MiniPrep Kit (Zymo Research, CA). First-strand cDNA was synthesized from 1 μg of total RNA using a qScript cDNA Synthesis Kit (Quanta Biosciences, MD). cDNA was used to perform RT-PCR using RT^2^ SYBR Green (Qiagen, CA) and GLUT8 primers (PPH13718A, Qiagen, CA). The cycling conditions consisted of 95°C for 10 minutes, followed by 40 cycles at 95°C for 30 seconds and 60°C for 1 minute. Relative quantification was used to calculate the difference between the target gene and the housekeeping gene 18S rRNA using the ΔC_T_ method [33].

#### Western blot analysis

Human placental samples were lysed in radioimmunoprecipitation assay buffer with a protease and phosphatase inhibitor cocktail (Thermo Fisher Scientific) on ice and centrifuged at 4°C. Supernatants were assayed for protein content using a BCA Protein Assay Kit (Thermo Fisher Scientific). Equal concentrations of samples, 20 μg of human placental tissue lysate protein were loaded onto 10% TGX gels (Bio-Rad, Hercules, CA) and subjected to gel electrophoresis. Human normal brain cerebellum tissue lysate (Abcam, Cat. # ab30069) was used as a positive control. The contents of the gels were transferred onto polyvinylidene difluoride (PVDF) membranes using Trans-Blot^®^ Turbo^™^ Transfer System (Bio-Rad). After blocking in 5% bovine serum albumin with 1× phosphate-buffered saline with Tween-20, membranes were incubated with primary antibody GLUT8 (Abcam, Cat. # ab169779) at 1:5000 dilution, and secondary antibody goat anti-rabbit IgG-horseradish peroxidase (Santa Cruz Biotechnology, Cat. # sc-2030) at 1:10,000 dilution. Vinculin (Sigma-Aldrich, Cat. # V4505) was used as a loading control at 1:30,000 dilution, and secondary antibody goat anti-mouse IgG-horseradish peroxidase was used at 1:10,000 dilution. Immunoreactive signals were analyzed using Pierce ECL Plus (Thermo Fisher Scientific) on a Typhoon Scanner 9410 (GE Healthcare Life Sciences, Pittsburgh, PA) through ImageQuant 5.2 software (GE Healthcare Life Sciences). The protein bands were quantified by densitometry using ImageJ software (National Institutes of Health, MD).

#### Immunohistochemistry

Serial sections of the placenta of 4 μm were cut in the same orientation from paraffin embedded tissue blocks and mounted onto Superfrost plus glass microscope slides. The slides were deparaffinized in xylene and rehydrated in descending concentrations of alcohol. Endogenous peroxidase activity was blocked with hydrogen peroxide in methanol for 10 minutes. Heat-induced antigen retrieval (HIER) was carried out for all sections in 0.01M Citrate buffer, pH = 6.00, using a Biocare decloaker at 95°C for 25 min. The slides were then cooled to RT, rinsed in PBS containing 0.05% Tween-20 (PBS-T), and incubated overnight at 4°C with anti-Humanin antibody (Sigma-Aldrich, Cat. # H2414) with negative control normal rabbit IgG (Santa Cruz, Cat. # sc-2027) at 1:50 and anti-GLUT8 antibody (Abcam, Cat. # ab169779) with negative control Normal rabbit IgG (Santa Cruz, Cat. # sc-2027) at 1:100 dilutions. They were then incubated for 1 hour at RT with anti-AE1/AE3 antibody (Dako, Cat. # M3515) with negative control normal mouse IgG (Santa Cruz, Cat. # sc-2025) at 1:500 dilution and rabbit polyclonal Lysozyme (Agilent, cat. #A0099) with negative control normal rabbit IgG (Santa Cruz, Cat. # sc-2027) at 1:1500 dilution. The sections were then rinsed with PBS-T. The signal was detected using the rabbit horseradish peroxidase EnVision kit (Dako, Cat. # K4003) for Humanin, GLUT8 and Lysozyme [34]. Mouse horseradish peroxidase EnVision kit (Dako, Cat. # K4001) was used for AE1/AE3 at RT for 30 minutes for detection. The sections were next rinsed with PBS-T and incubated with DAB (3,3’-Diaminobenzidine) for visualization. The sections were washed in H2O, counterstained with Harris’ Hematoxylin, dehydrated in EtOH, and cover slipped with mounting media.

#### Fluorescent immunohistochemistry

Non-specific binding was blocked on deparaffinized slides with 0.2% Triton X-100 in 5% normal donkey serum and 1% gelatin at room temperature (RT) for 1 hour. Sections were then incubated with anti-GLUT8 antibody (abcam, Cat. # ab169779) at 1:20 and negative control normal rabbit IgG (Santa Cruz, Cat. # sc-2027) at 1:20 at RT for 2 hours, followed by overnight incubation at 4°C. Sections were washed thoroughly with 1x PBS and incubated with Texas red-conjugated donkey anti-rabbit secondary antibody (Jackson Immunoresearch, Cat. # 711-295-152) at 1:500 and DAPI (4’,6-diamidino-2-phenylindole dihydrochloride) antibody (Sigma, Cat. #D9542) at 1:1000 at RT for 1 hour. The images were captured under a Nikon E-600 microscope (Nikon, NY), equipped with a cooled charge-coupled device (CCD) camera (Cool SNAP HQ Monochrome, Roper Scientific, AZ). To quantify GLUT8 expression, fluorescence images were observed. There were 5 control and 5 IUGR placentas. There was 1 full thickness section for each placenta. 17–22 fields were randomly selected for each section. Images were acquired at 12-bit gray level resolution and displayed in pseudocolor (monochrome) for analysis with GLUT8 represented by Texas red-conjugated donkey anti-rabbit secondary antibody (Jackson Immunoresearch, Cat. # 711-295-152). The Metamorph image analysis system software (Molecular Devices, CA) was used to measure integrated optical density for GLUT8 expression.

HTR8/SVneo cells were also fixed in 4% paraformaldehyde and washed with PBS. Cells were incubated overnight at 4°C with Anti-Humanin antibody (Sigma, Cat. #H2414) at 1:300 and Anti-GLUT8 antibody (Abcam, Cat. # ab169779) at 1:10 dilution. Normal rabbit IgG (Santa Cruz, Cat. # sc-2027) was used with GLUT8 and Humanin as negative controls. The fixed cells were next rinsed with PBS-T and subsequently incubated with a mixture of Alexa Fluor 488-conjugated donkey anti-rabbit IgG (Jackson Immunoresearch, Cat. #711-545-152) at 1:250 and DAPI (Sigma, Cat. #D9542) at 1:1000 in RT for 1 hour. The Nikon E-600 microscope mounted with a cooled charge-coupled device (CCD) camera (CoolSnap HQ Monochrome) was utilized to capture images under a fluorescence microscope at 40x magnification.

#### Immunoprecipitation (IP) assay

Immunoprecipitation was performed using Dynabeads protein G Immunoprecipitation kit (Invitrogen, Vilnius, LT). 50ulit of Dynabead first incubate with 10ugm of humanin (Sigma H2414) and GLUT8 (Ab 169779) separately with rotation at room temperature for 2hrs. Rabbit non-isotype IgG was used as negative control. The beads were then separated on the magnet and supernatant was removed. The beads–Ab complex were washed in PBS with Tween 20, then 300ugm of protein lysate were added to the washed beads and allow them to bind the antigen with Dynabeads–Ab complexes overnight at 4°C with rotation. Next the beads were separated by magnet and washed the Dynabeads Ab-Ag complexes using washing buffer, finally the beads were boiled with 4xSDS sample loading buffer at 95°C for 5 minutes, and the samples were analyzed by western blot.

Human primary villous trophoblast cells (ScienCell, Cat. # 7120) were cultured in trophoblast medium (ScienCell, Cat. #7121) with trophoblast growth supplement (ScienCell, Cat. #7152) and 1% penicillin-streptomycin solution (ScienCell, Cat. #0503) in an incubator with an atmosphere of 5% CO2/95% air. The cells were cultured in the media both with 5% fetal bovine serum and without fetal bovine serum for 8 hours and 24 hours. After 8 and 24 hours, cells were fixed in 4% paraformaldehyde and washed with PBS. Cells were incubated overnight at 4°C with anti-Humanin antibody (Sigma, Cat. #H2414) at 1:300 and anti-GLUT8 antibody (Abcam, Cat. # ab169779) at 1:10 dilution. Normal rabbit IgG (Santa Cruz, Cat. # sc-2027) was used for GLUT8 with Humanin as negative control. The fixed cells were next rinsed with PBS-T and subsequently incubated with a mixture of Alexa Fluor 488-conjugated donkey anti-rabbit IgG (Jackson Immunoresearch, Cat. #711-545-152) at 1:250 and DAPI (Sigma, Cat. #D9542) in RT for 1 hour. The Nikon E-600 microscope mounted with a cooled charge-coupled device (CCD) camera (CoolSnap HQ Monochrome) was utilized to capture images under a fluorescence microscope at 40x magnification. 2–4 fields were randomly selected for each sample. Images were acquired at 12-bit gray level resolution and displayed in pseudocolor (monochrome) for analysis with GLUT8 and Humanin represented by Alexa Fluor 488-conjugated donkey anti-rabbit IgG (Jackson Immunoresearch, Cat. #711-545-152). The Metamorph image analysis system software (Molecular Devices, CA) was used to measure integrated optical density for GLUT8 and Humanin expression.

### Data analysis

For mRNA and Western blot analyses, the integrated band density readout in arbitrary densitometric units was normalized based on control samples (i.e. value of 100%) for each experimental set of conditions. Statistical analysis was performed using SigmaStat 3.5 and StatView 5.0 softwares. We checked normality assumption of the raw data and if it was not violated, we used Student’s *t* test (if 2 groups) or ANOVA (if >2 groups) in group comparisons. When more than 2 groups were involved in the comparison and there was an overall statistically significant difference, we did pairwise comparisons using Tukey’s type I error (set at 5%) adjustment method in order to control for the inflation of Type I error. Type I error rate can inflate when you do multiple comparisons (ie pairwise comparisons). If the data was not normally distributed, we used nonparametric methods such as Wilcoxon rank sum test (Student t test analog) or Kruskal Wallis teat (ANOVA analogue). In testing the main effects of time and treatment and time*treatment interaction, we performed 2-way ANOVA incorporating the interaction term. All data are shown as mean±SEM, and statistical significance was assigned at p<0.05. Different p-value cutoff scores were indicated as following: * = p<0.05, ** = p<0.01, *** = p<0.001, and **** = p<0.0001.

## Results

### mRNA and protein levels of GLUT8 are increased in IUGR-affected pregnancies

**Fig 1:**
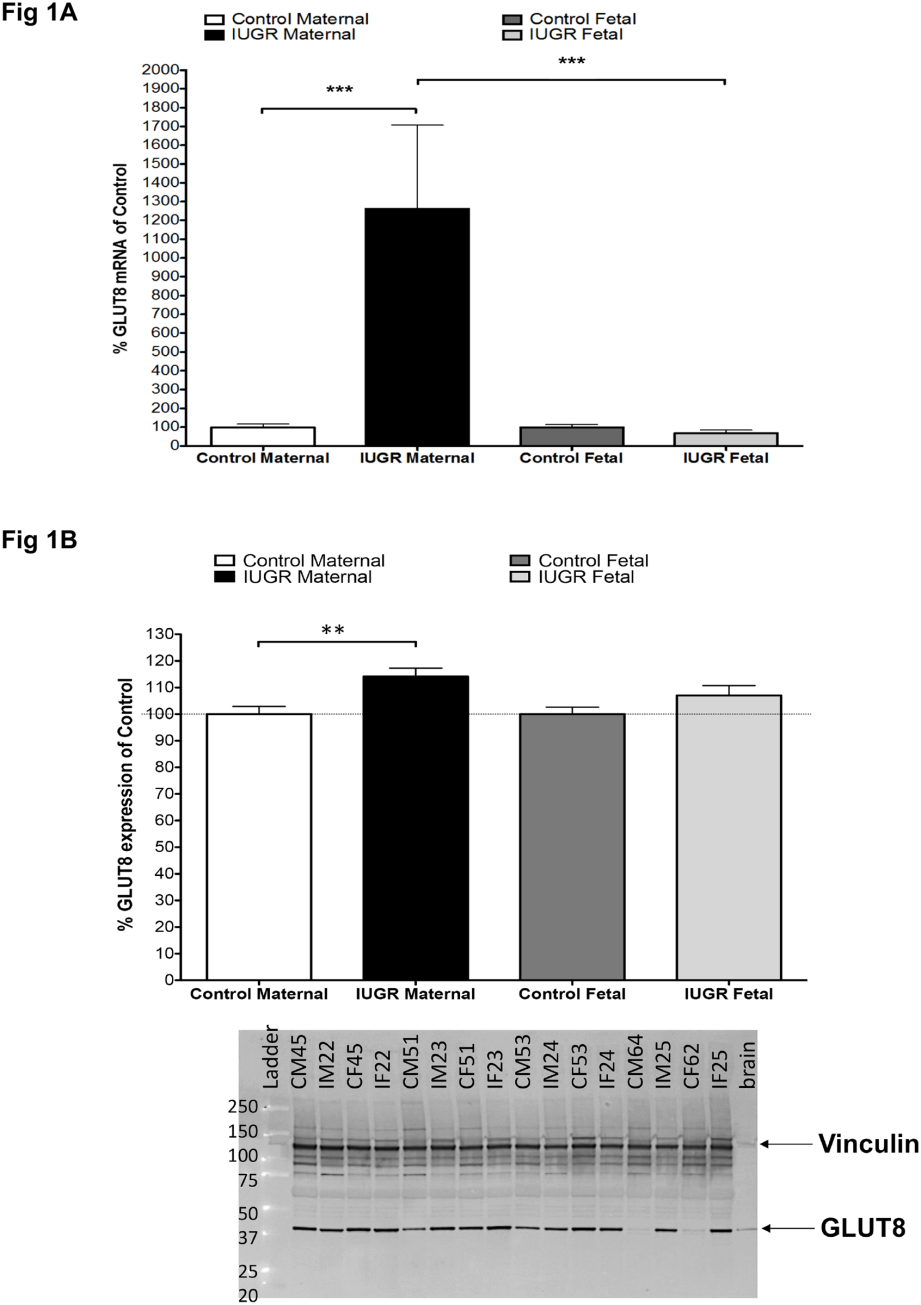
mRNA and protein levels of GLUT8 are increased in IUGR-affected pregnancies. (**A**) Real-time quantitative RT-PCR analysis of placental GLUT8 expression. IUGR-affected placentas (n = 22) were assayed against gestational age-matched, control placentas (n = 22) for the maternal and fetal sides. Relative quantification of PCR products was based on the C_T_ value differences between target and the housekeeping gene using the comparative C_T_ method (Eukaryotic 18S rRNA) was used as an internal control. (*** = p<0.001). (B) Western blot analysis of GLUT8 in placental biopsies obtained from women with normal pregnancy (n = 22) or IUGR-affected pregnancy, by abnormal prenatal ultrasound (n = 22). Vinculin was used as the loading control. Representative blots are shown. (** = p<0.01). The arrow pointing at the dark band at approximately 130 kDa indicates Vinculin. The arrow pointing at the dark band at approximately 51 kDa indicates GLUT8. Sample label abbreviations are as follows: "CM" is control maternal side, "CF" is control fetal side, "IM" is IUGR maternal side, and "IF" is IUGR fetal side. The number following the sample abbreviation indicates sample ID. The same number on multiple labels indicates the same sample. Brain was used as a positive control for GLUT8.

mRNA and protein expression of GLUT8 on the fetal and maternal aspects of the placenta from IUGR-affected and normal pregnancies are shown in Fig 1. *GLUT8* mRNA levels were increased significantly by nearly 13-fold in the IUGR samples on the maternal aspect of the placenta versus control samples (Fig 1A). A significant difference in *GLUT8* mRNA expression between maternal and fetal IUGR-affected placentas was detected as well. GLUT8 protein expression was also increased on the maternal face of the placenta from IUGR-affected pregnancies compared to normal (Fig 1B). There were no appreciable differences observed on the fetal side of the placenta.

**Fig 2:**
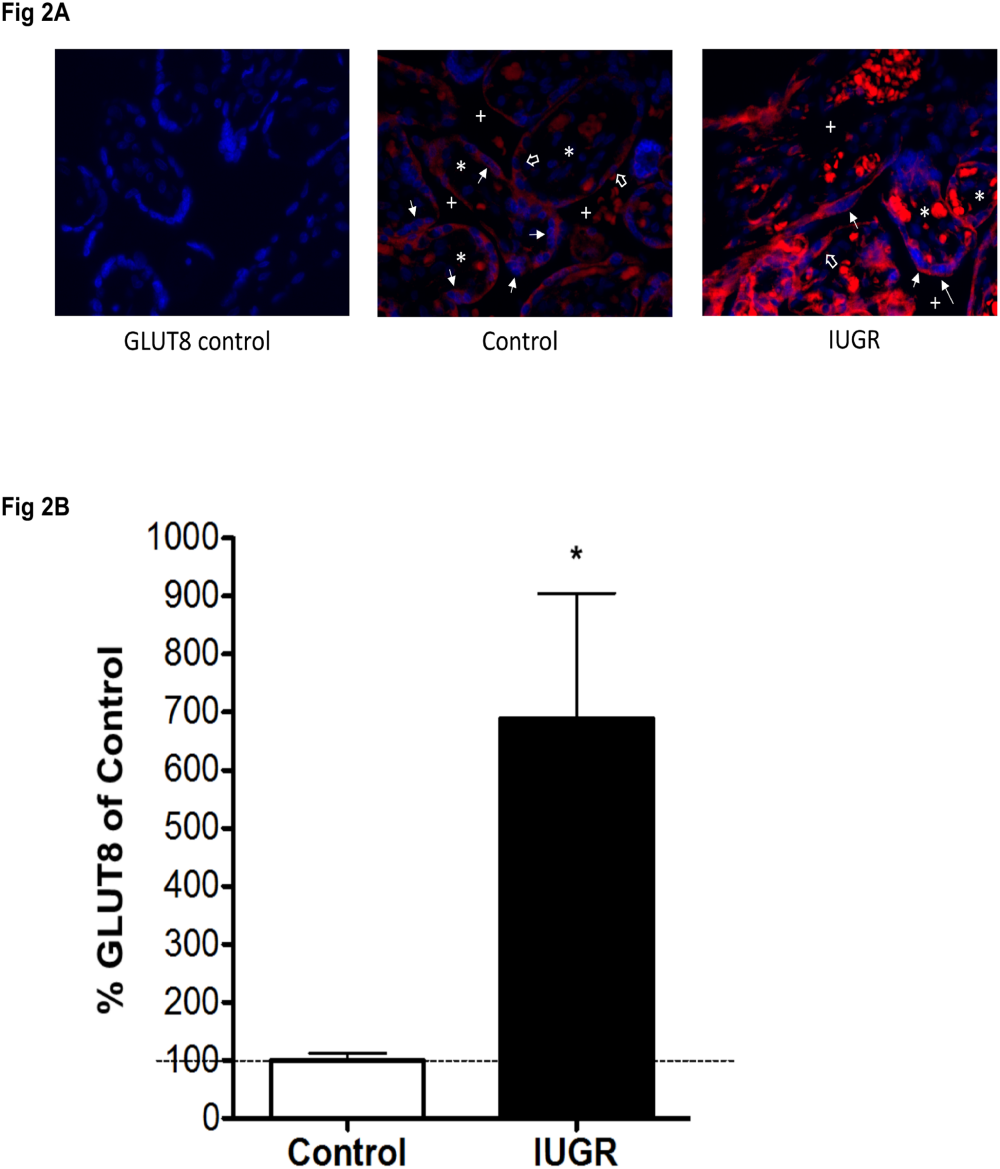
Increased immunofluorescence of GLUT8 in IUGR-affected placentas. (A) Immunoflourescence staining of GLUT8 (red) and DAPI (blue) of placental tissue. Left: negative control performed with non-isotype IgG to measure the level of non-specific background signal. Middle: GLUT8 staining in the normal placenta. Right: GLUT8 staining of the IUGR-affected placenta. Plus sign (+) indicates intervillous space. Asterisk (*) shows villous core. Line arrows show syncytiotrophoblast. Hollow arrows show cytotrophoblast. All images taken at 40X magnification. (B) Relative quantitation of GLUT8 immunofluorescence staining of GLUT8 in the normal and IUGR-affected human placenta. (* = p<0.05). Images were captured with Nikon E-600 microscope (Nikon, NY), equipped with a cooled charge-coupled device (CCD) camera (Cool SNAP HQ Monochrome, Roper Scientific, AZ). There were 5 control and 5 IUGR placentas, full thickness section for each placenta. 20 fields were randomly selected for each section. Images were acquired at 12-bit gray level resolution and displayed in psuedocolor (monochrome) for analysis with GLUT8 represented by Alexa Fluor 488-conjugated donkey anti-rabbit IgG. The Metamorph image analysis system software (Molecular Devices, CA) was used to measure integrated optical density for GLUT8 expression.

GLUT8 immunofluorescence staining was subsequently performed in human IUGR-affected and control placental samples to visualize GLUT8 cellular localization and expression differences. Fig 2 shows increased expression of GLUT8 in IUGR-affected placenta by immunofluorescence. Quantitation of the staining shows a 7-fold elevation in the expression of GLUT8 protein in the IUGR-affected placenta compared to control seen in Fig 2B.

### HN expression is increased in IUGR-affected pregnancies

**Fig 3:**
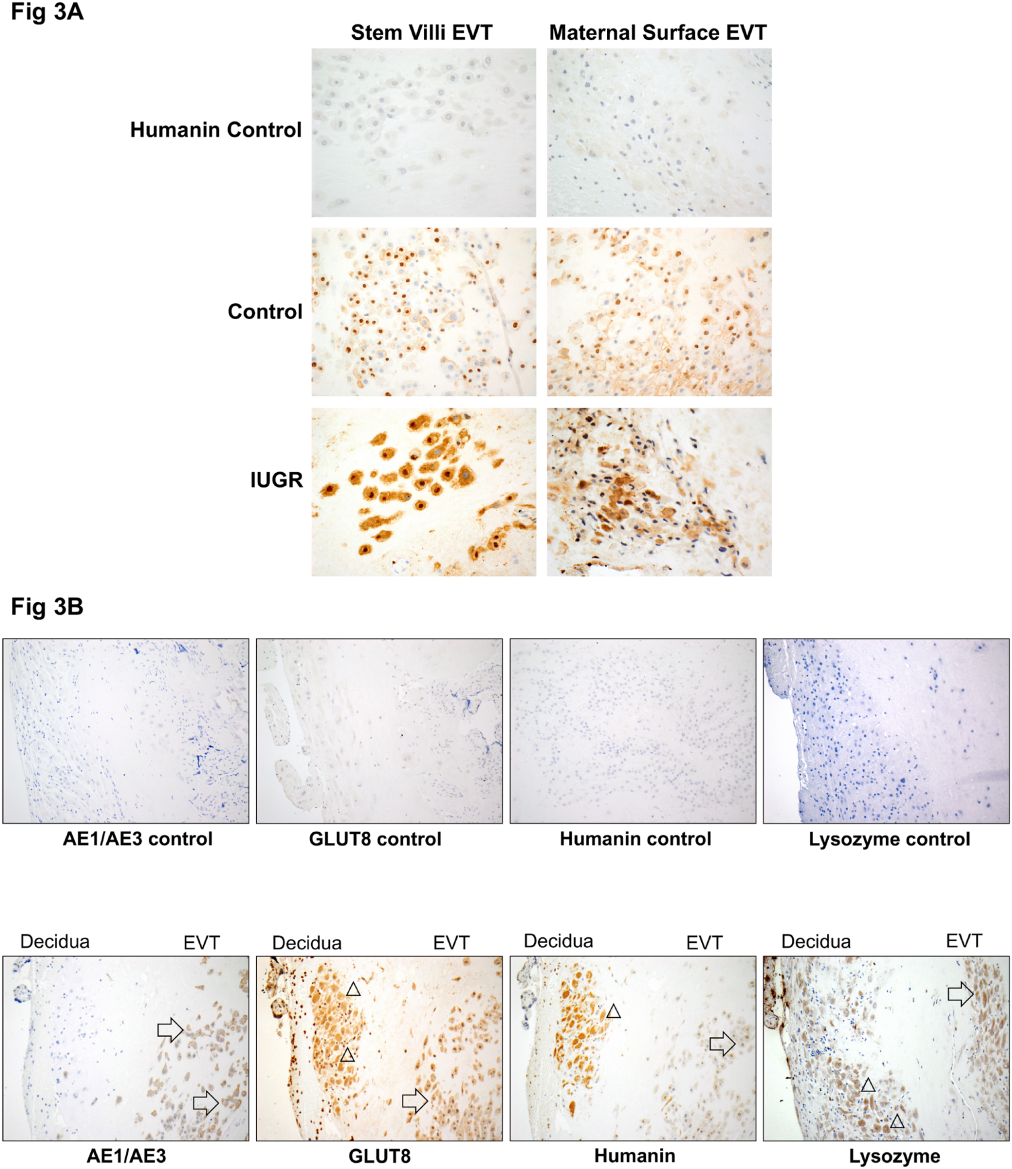
Increased immunohistochemical staining of HN in IUGR-affected placentas. (A) Immunohistochemical staining of humanin (HN) in human placental tissue at gestational age of 38 weeks. Humanin was stained in brown. The left column shows staining of the stem villi EVT of placental tissue, and the right column shows staining of the maternal surface EVT of placental tissue. The top row of both columns is negative control performed with non-isotype IgG. The middle row of both columns is placenta from normal pregnancies. The bottom row of both columns is IUGR. 40x magnification for all images. (B) Immunohistochemical staining in normal (control) human placental tissue showing four different markers of same placental sample (serial sections) at 38 weeks gestational age. The top row is the non-isotype IgG control for each of the markers labeled. The bottom row from left to right is staining of AE1/AE3 (trophoblast marker), GLUT8, Humanin, and lysozyme (lysosomal marker). The presence of EVT on the right portion of each image is supported by diffuse cytoplasmic staining of AE1/AE3, while the endometrial stromal cells within the decidua on the left portion of each image are negative for AE1/AE3. Each of the four images on the bottom row shows the decidua on the left side and maternal surface EVT on the right side of the image. Arrows point to maternal surface EVT and triangles indicate decidual stromal cells. All images were taken at 20x magnification.

We next performed immunohistochemical staining in human placenta to determine HN expression. We did not measure mRNA levels of HN since the location of the gene (or genes) encoding the HN peptide has not been determined conclusively [35]; there are more than 10 putative transcription sites for the endogenous HN peptides encoded by the *MT-RNR2*-like nuclear genes. Fig 3A shows that HN is expressed in extravillous trophoblasts (EVTs) in the maternal decidua and also in EVTs on the maternal surface of the placenta. This expression was increased in IUGR-affected placenta compared to control. Fig 3B shows HN and GLUT8 expression in serial sections of a control placenta (38 weeks). In order to label trophoblast cells (EVT in the decidua and the maternal surface of the placenta), we used anti-cytokeratin cocktail AE1/AE3 (left panel). There is significant overlap between the cells (trophoblasts) that stain positive for both HN and GLUT8. Cell staining with lysosomal marker reveals granular cytoplasmic staining of decidual stroma and maternal surface EVTs in a similar distribution of trophoblast cells.

### *In vitro* trophoblast cell studies

#### Confirmation of HN and GLUT8 interaction

**Fig 4:**
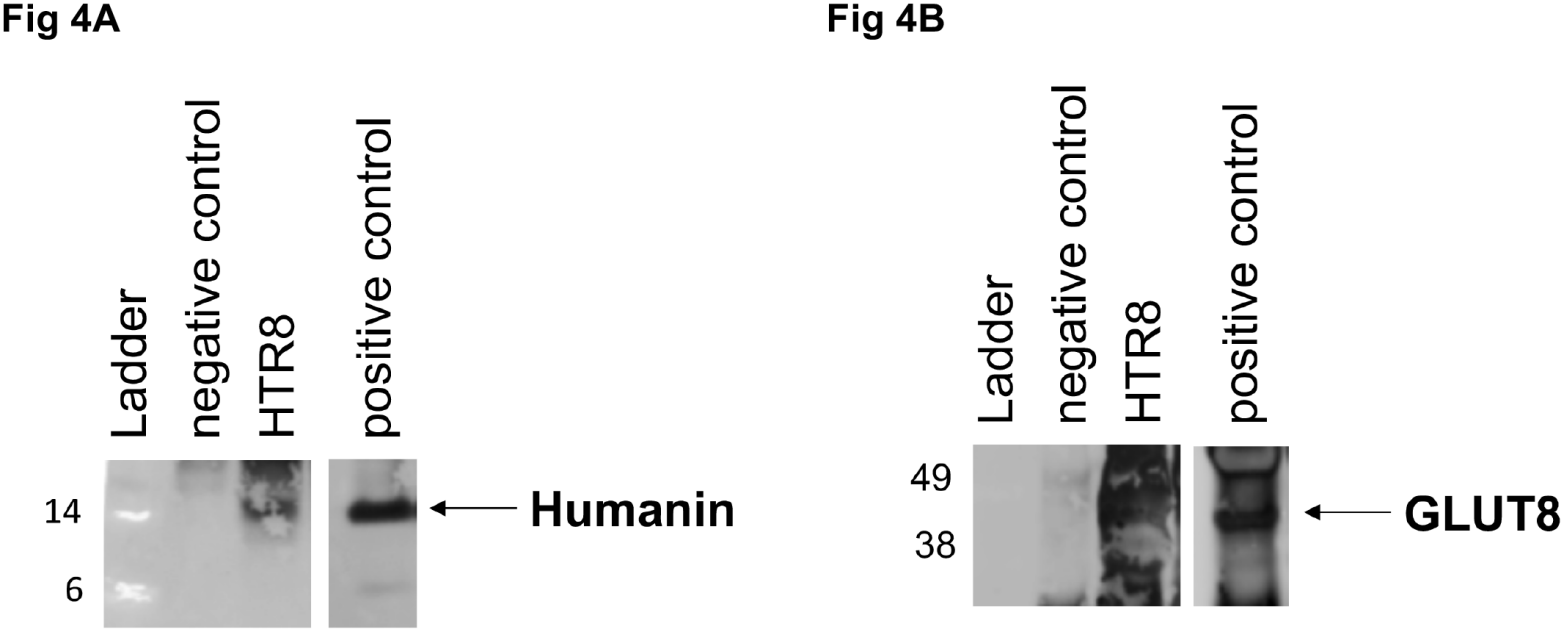
Immunoprecipitation (IP) assays reveal association between endogenous humanin with Glut8 in HTR-8 cells. (A) HTR8/SVneo protein was immunoprecipitated with Glut8 antibody then the bound protein was analyzed by western blot with humanin antibody. (B) HTR8/SVneo protein lysate was immunoprecipitated with humanin antibody then analyzed by western blot with glut8 antibody. In both the cases Rabbit non-isotype IgG was used as negative control (-C) and in positive control (+C), both the immunoprecipitation and western blot analyses were done using the same antibody (either humanin or Glut8 antibody).

In order to study whether HN and GLUT8 are proteins that interact *in vivo*, we performed coimmunoprecipitation (coIP) in a well-established human EVT cell line, HTR8/SVneo human trophoblasts. Fig 4 shows that coIP of HN and GLUT8 confirmed an *in vitro* interaction. First endogenous GLUT8 protein was immunoprecipitated from HTR8/SVneo cells with either non-istotype IgG (negative control) or GLUT8 antibody (left panel). The precipitates were subjected to western immunoblot analysis with anti-HN antibody. As shown in Fig 4A, humanin precipitates with GLUT8. Inversely, GLUT8 precipitates with HN (Fig 4B).

#### Cytoplasmic localization of HN and GLUT8 in human extravillous trophoblastic cells

**Fig 5:**
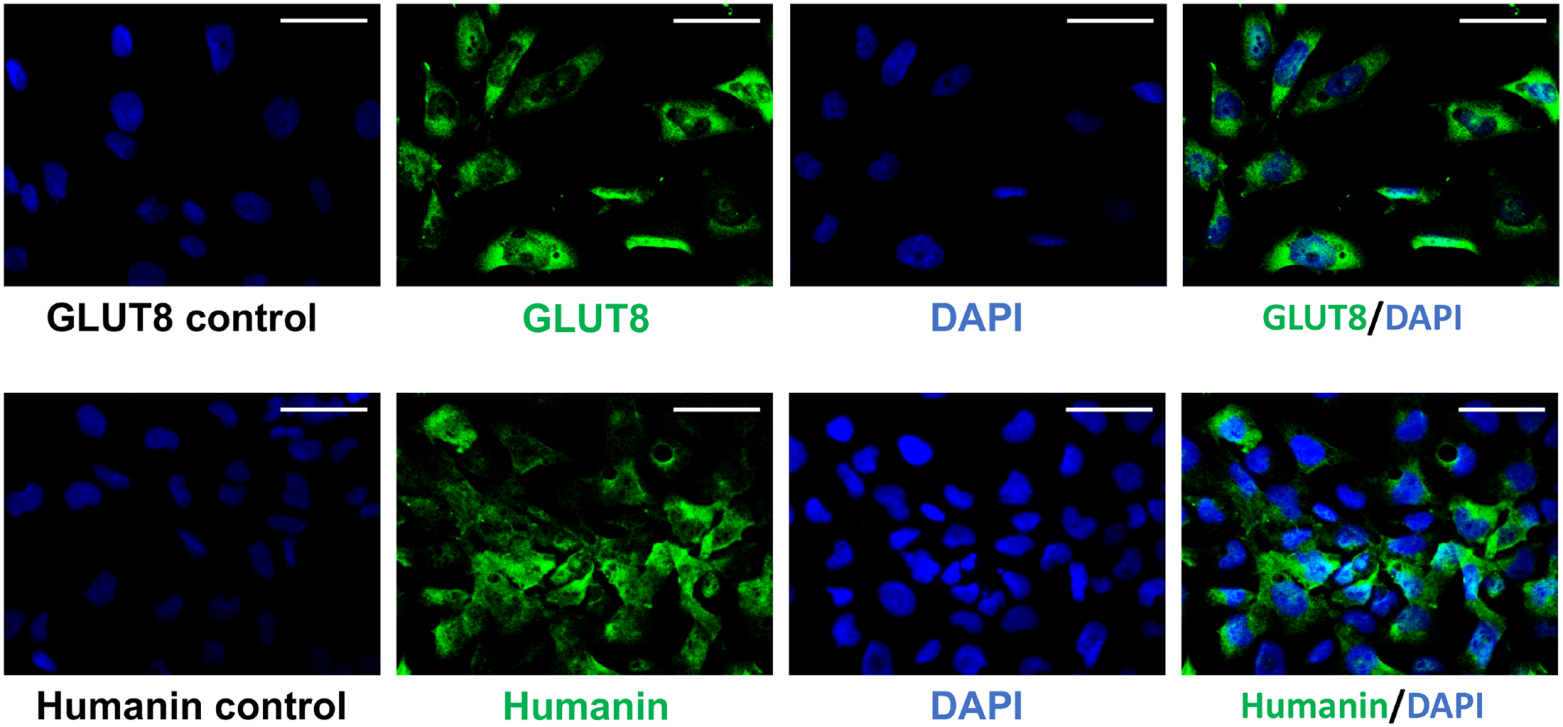
Immunocytochemical staining showing localization of HN and GLUT8 in HTR8/SVneo cells. The top rows show localization of GLUT8. On the top row, the left image is the non-isotype IgG control, the left middle is GLUT8 (green) staining taken using a filter for GLUT8 alone, the right middle is DAPI (blue) taken using a filter for DAPI alone, and the right image is GLUT8 (green) and DAPI (blue) combined. The 3 rightmost images are of the same area. The bottom rows show images of humanin. On the bottom row, the left image is the non-isotype IgG control, the left middle is Humanin (green) staining using a filter for Humanin alone, the right middle is DAPI (blue) using a filter for DAPI alone, and the right image is Humanin (green) and DAPI (blue) combined. The three rightmost images are of the same area. All images have 40x magnification. Scale bar = 50 μm.

To determine nuclear vs. cytoplasmic staining of HN and GLUT8 in human trophoblasts, we performed immunohistochemistry in HTR8/SVneo cells (Fig 5). Two representative fields (A and B) of untreated cells are shown at 40x magnification. Fig 5A shows similar immunolocalization of HN (green) and DAPI (blue), and Fig 5B shows similar immunolocalization of GLUT8 (green) and DAPI (blue). HN and GLUT8 both positively stained predominantly in the cytoplasm. This result supports localization of HN in the human placental system.

#### Increased HN and GLUT8 expression in HTR8/SVneo cells with CoCL_2_

**Fig 6:**
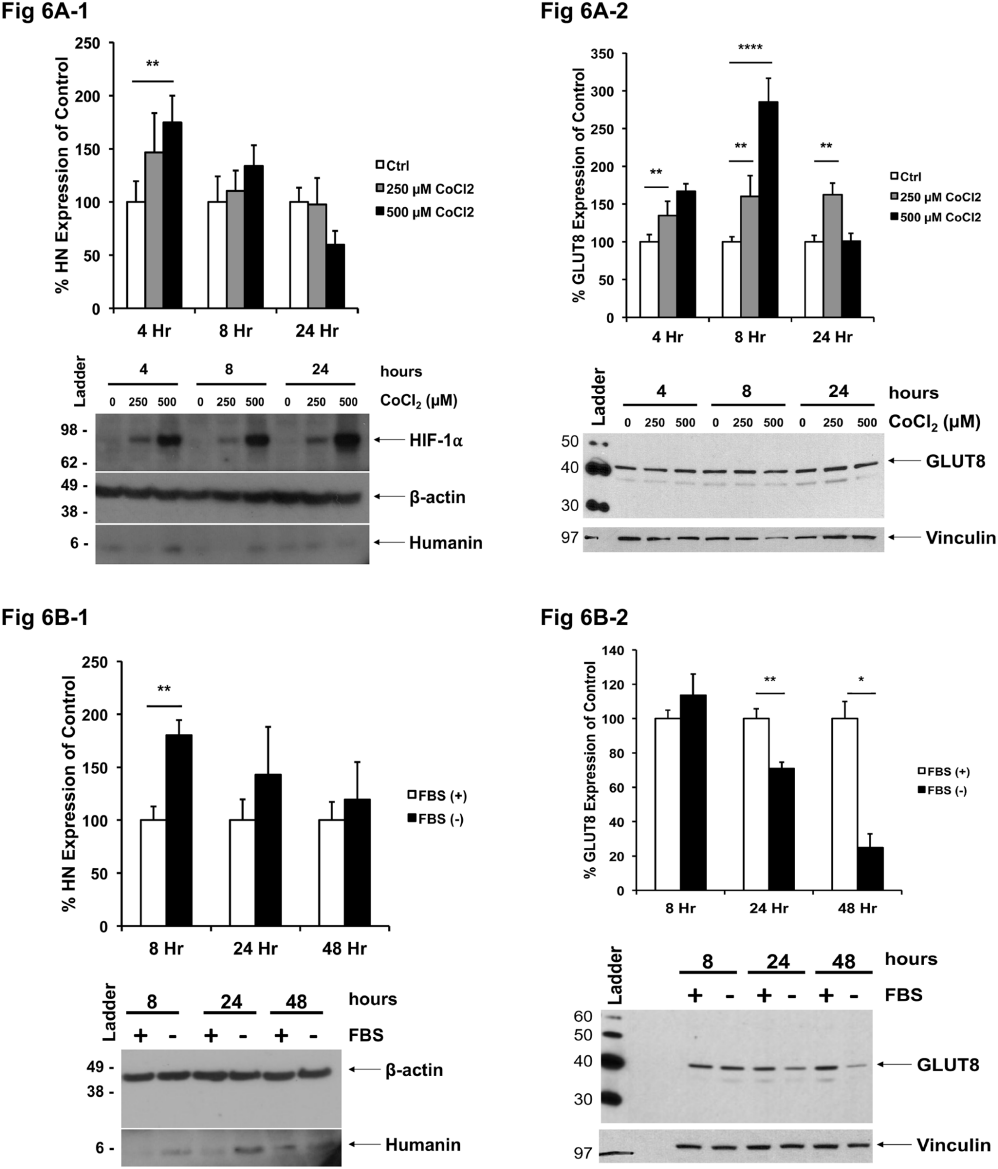
CoCl_2_-stimulated hypoxia induces HN and GLUT8 expression in HTR8/SVneo cells, and serum-deprived HTR8/SVneo cells up-regulates HN levels but down-regulates GLUT8 levels. (**A**) HN and GLUT8 levels were determined by Western blot analysis at 4, 8, and 24 hours of incubation of HTR8/SVneo cells in 250 and 500 μM CoCl_2_–supplemented complete media. Representative blots are shown. β-actin and vinculin were used as loading controls for HN and GLUT8, respectively (n = 3, * = p<0.05, ** = p<0.01, and *** = p<0.001). (**B**) HN and GLUT8 expression was also monitored in HTR8/SVneo cells at 8, 24 and 48 hours of cultivation in serum-supplemented (FBS (+)) and serum-free media (FBS (-)). Representative blots are shown. β-actin and vinculin were used as the loading controls for HN and GLUT8, respectively. (n = 3, * = p<0.05 and ** = p<0.01).

In order to simulate conditions of hypoxic stress, we administered, cobalt chloride (CoCl_2_), a chemical mimetic of hypoxia [32], to HTR8/SVneo cells, in order to observe the effect on the expression of HN and GLUT8 (Fig 6A). Hypoxia was confirmed by heightened expression of hypoxia inducible factor-1α (HIF-1α). Incubation of human EVT cells with 500 μM CoCl_2_ for 4 hours resulted in a peak 1.7-fold induction of HN with decreasing levels by 24 hours (Fig 6A-1). GLUT8 levels increased after incubation with CoCl_2_, which is used to mimic hypoxic conditions, with a delayed peak of a 2.9-fold rise after 8 hours with decreasing levels after 24 hours (Fig 6A-2). Therefore, CoCl_2_ addition results in a time and dose-dependent elevation in the levels of GLUT8 with an overall delayed and prolonged effect compared to the changes in HN expression under the same experimental conditions.

#### HTR8/SVneo cells under serum starvation experience up-regulation of HN but attenuation of GLUT8 expression

Growth factor deprivation in HTR8/SVneo cells was also considered to examine another condition associated with IUGR. This condition was mimicked by treatment with serum-free growth medium, and these cells were compared with their serum-supplemented (10% FBS) counterparts at 8, 24, and 48 hours (Fig 6B). There was a 1.8-fold increase in HN expression after 8 hours of serum deprivation compared to controls (Fig 6B-1). Conversely, serum deprivation results in a significant reduction in GLUT8 expression—one-third (29%) and 4-fold after 24 hours and 48 hours, respectively, compared to control groups (Fig 6B-2). Thus, unlike CoCl_2_ treatment, serum deprivation has a delayed effect and results in decreased GLUT8 expression.

#### Evaluation of GLUT8 and HN expression in primary human trophoblasts exposed to serum starvation

**Fig 7:**
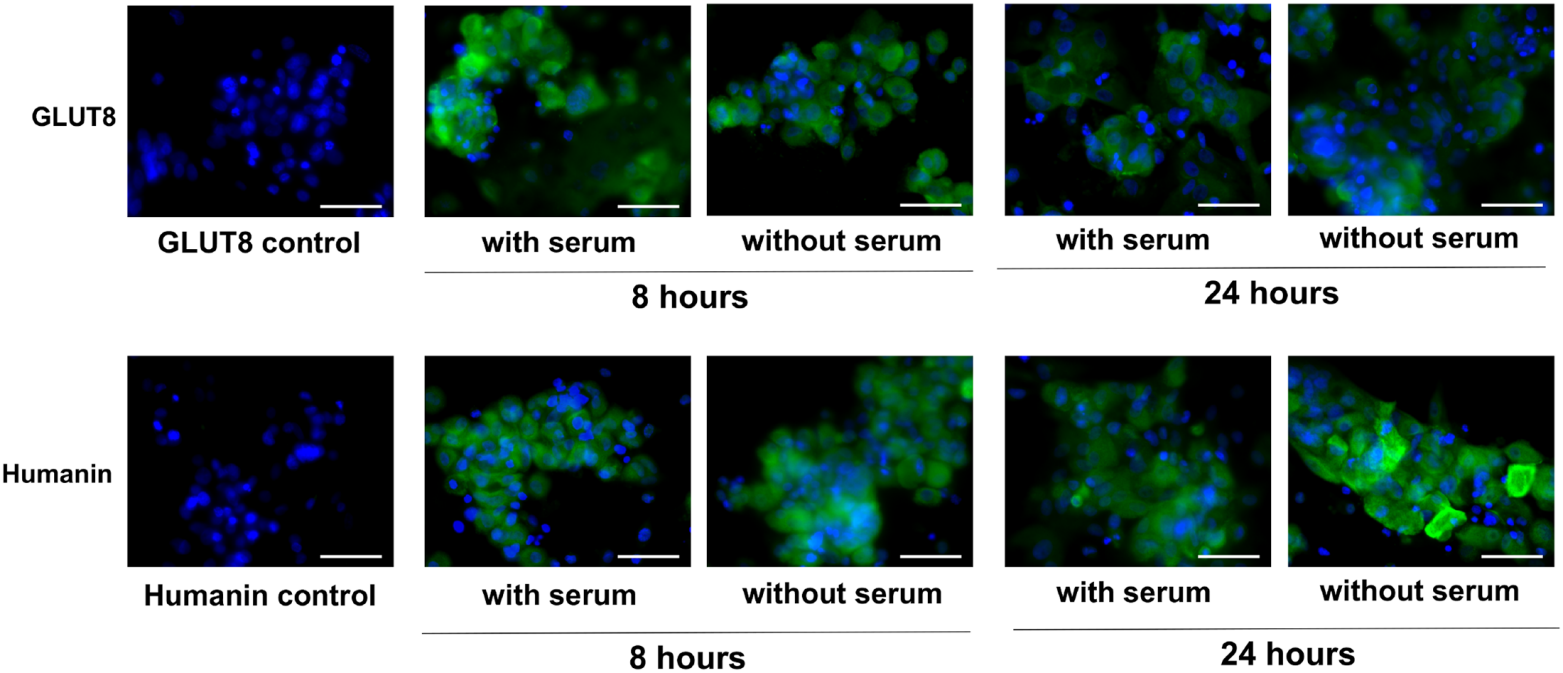
Primary human trophoblasts exposed to serum starvation. Top row is GLUT8 staining (green) of human primary villous trophoblast cells. Bottom row is humanin staining (green) of human primary villous trophoblast cells. All images show DAPI staining of nuclei (blue). From left to right: non-isotype IgG control, cells grown for 8 hours with serum, cells grown for 8 hours without serum, cells grown for 24 hours with serum, cells grown for 24 hours without serum. All images taken at 40x magnification. Scale bar = 50 μm.

To examine the effect of growth factor deprivation on the expression of HN and GLUT8, we grew primary human trophoblasts in culture with and with out serum for 24 hours. Representative photomicrographs with HN and GLUT8-specific fluorescence are shown in Fig 7. Similar to HTR8/SVneo cells, we found with serum deprivation, there was a trend towards increased HN expression but decreased GLUT8 without serum at 8 and 24 hours.

## Discussion

In this study we show, for the first time, that HN is expressed in the human placenta, and that there is increased HN and GLUT8 expression on the maternal aspect of the placenta in pregnancies affected by idiopathic IUGR. HN has demonstrated cytoprotection in multiple aging-related and neurologic disease processes, including Alzheimer’s disease [9, 36, 37], conditions of memory loss [38], cardiovascular disease [6, 39], type 1 diabetes [40], and stroke [41]. A pathophysiologic mechanism common to many of these diseases is oxidative stress, which also occurs during normal and abnormal pregnancies [42–46]. A physiological level of oxidative stress and nutrient transport is required for proper embryonic and fetal growth [45]. However, an altered degree of oxidative stress and nutrient exchange in the intrauterine environment becomes problematic and is linked to complications of pregnancy such as maternal vascular disease, gestational diabetes, placental insufficiency, and pre-eclampsia [3]. In particular, increased oxidative stress induced by hypoxia-ischemia is suggested to lead to IUGR of the developing fetus and associated metabolic diseases subsequently throughout life [1–3].

Many studies on exogenous administration of HN have shown that it mitigates oxidative stress-associated apoptosis and improves tissue functions in non-placental systems [5–8, 47]. No previous investigations, however, have examined HN expression in human placentas. Interestingly, a recent study has shown elevated levels of HN in the human sera of pre-eclamptic pregnancies versus normal pregnancies, suggesting a role of HN during pregnancy [15]. These studies provided the rationale to examine HN in placental pathology.

Conversely, GLUT8 is a dual-specificity glucose and fructose transporter, which varies in its localization and function depending on the tissue in which it is expressed [27, 48]. GLUT8 participates in insulin-stimulated cell-membrane translocation from an intracellular vesicular compartment, for hexose transport only in the blastocyst [27]. In other cell types including the brain [49, 50], however, GLUT8 is involved exclusively in intracellular hexose transport with no evidence of membrane localization [51]. This tethering of GLUT8 within intracellular vesicles is due to the presence of a di-leucine motif, abrogation of which lends the ability to translocate to the plasma membrane [50]. This intracellular organelle location of GLUT8 may be responsible for fueling the mitochondrial machinery and interplay with HN, since this cytoplasmic location of GLUT8 closely mimics the HN localization described previously in human skeletal muscles [52]. Despite a large number of studies on HN and GLUT8, no studies have examined the co-localization of HN and GLUT8 in human IUGR-associated conditions. Therefore, our study examined both HN and GLUT8 expression simultaneously in an attempt to determine their relationship in normal and IUGR-affected placentas.

Our results show that the human placenta affected by idiopathic late-term IUGR exhibits variable expression of GLUT8, which is increased on the maternal aspect of the placenta, but unchanged on the fetal aspect of the placenta. These results are similar to our previous findings for GLUT3 [26], which also showed a similar expression profile in IUGR. Increased placental GLUT8 levels were associated with increased HN expression levels, suggesting that HN and GLUT8 may share a similar regulatory mechanism for expression in IUGR. Both HN and GLUT8 demonstrated cytoplasmic localization to extravillous trophoblasts (EVTs) on the maternal aspect. Given this localization pattern, we also evaluated isolated EVTs *in vitro* and found that both HN and GLUT8 displayed similar localization patterns with cytoplasmic distribution of these two proteins. To evaluate interaction between HN and GLUT8, we next undertook co-immunoprecipitation experiments, revealing protein-protein binding, suggesting a role for HN and GLUT8 working together.

To further pinpoint the inciting factor(s) that up-regulate(s) HN in human IUGR-affected placentas, we next employed *in vitro* strategies with HTR8/SVneo human EVT cell cultures. Since EVT plays a key role in early implantation events, we subjected them to two non-physiologic, oxidative stress-associated conditions that have been correlated with IUGR [53, 54]: (1) persistent placental hypoxia (associated with poor trophoblast invasion and reduced utero-placental perfusion [2, 3], and (2) maternal growth factor restriction (associated with diminished trans-placental glucose transport and gene expression profiles seen in IUGR phenotypes) [55]. Under these conditions, EVTs demonstrate a cytoplasmic localization of both HN and GLUT8. However, their protein expression profiles displayed up-regulation of HN and GLUT8 under chemically stimulated hypoxia, but only HN expression was elevated in growth factor restriction while GLUT8 expression was reduced. Thus, one may surmise that hypoxia may be necessary for inducing GLUT8, but growth factor restriction or hypoxia alone both up-regulate HN. Therefore, our current finding of increased HN and GLUT8 in IUGR-affected placental EVTs suggests a major role for uteroplacental insufficiency-induced hypoxia as the inciting factor. Both HN and GLUT8 have previously been induced under *in vitro* hypoxic conditions [16, 17] in other cell types, lending credence to our present *in vivo* observations. The time-dependent studies revealed that HN expression elevation occurred acutely at 4 hours of hypoxia while GLUT8 increased at 8 hours, subsequent to the change in HN. This observation supports the paradigm that HN increase precedes the GLUT8 increase that may be necessary for the biologic role of GLUT8 in transporting intracellular glucose to organelles necessary to fuel mitochondrial energy metabolism. In addition to the EVTs alone, primary cultures of human trophoblasts also revealed a similar effect of serum starvation, suggesting a similar effect beyond the EVTs alone. Studies of the subcellular localization of GLUT8 indicate that GLUT8, found on the membrane of the endoplasmic reticulum (ER), may play a role in glucose transport between the cytosol and intracellular organelles [51]. Indeed, in GLUT8 knockout mice, GLUT8 deletion resulted in impaired mitochondria function in sperm cells and a 50% decrease in ATP production [56]. Further, GLUT8 deletion (-/-) caused a reduction in murine litter size and pup size due to impaired decidualization and implantation [57], supporting a functional role for the presence of GLUT8 in extravillous trophoblasts.

Although several papers have already shown HN localization in tissues such as hippocampus [36], skeletal muscles [52], and testes [58], this original article presents for the first time the presence of endogenous HN in human placenta. Our present observations are also the first-of-a-kind to examine interplay between placental HN and GLUT8 and demonstrate a potential relationship given their similar sub-cellular localization, protein-protein interaction, and expression. We speculate, based on the data presented in this report, that HN and GLUT8 play a protective role in governing the physiological adaptations required of the IUGR-affected placenta to ensure survival within an adverse intrauterine environment. Further studies are necessary to elucidate their physical interactions and the molecular and cellular mechanisms that govern these interactions in normal and IUGR-affected placentas.

### Conclusions

In summary, we have established the sub-cellular cytoplasmic presence of HN and GLUT8 in human placental EVTs found on the maternal aspect, and demonstrated an up-regulation in IUGR. In addition to this novel finding, we have established with the help of *in vitro* EVT cell cultures, that while simulated hypoxia regulates both HN and GLUT8, only growth factor restriction increased HN. Most importantly a protein-protein interaction between HN and GLUT8 suggests that their interaction may fulfill a biologic role that requires interdependency. Future investigations delineating molecular interactions between these proteins are required to fully uncover their role in IUGR-affected pregnancies.

## Supporting information

S1 Fig Original western blots. Original uncropped and unadjusted western blots used to create Fig 6 in manuscript are provided here as labeled.
(TIF)
