# Supplementary figures and images for: Humanin (HN) and glucose transporter 8 (GLUT8) in pregnancies complicated by intrauterine growth restriction
Source: PLoS One. 2018 Mar 28;13(3):e0193583. doi: 10.1371/journal.pone.0193583 (PMC5873989; doi:10.1371/journal.pone.0193583)

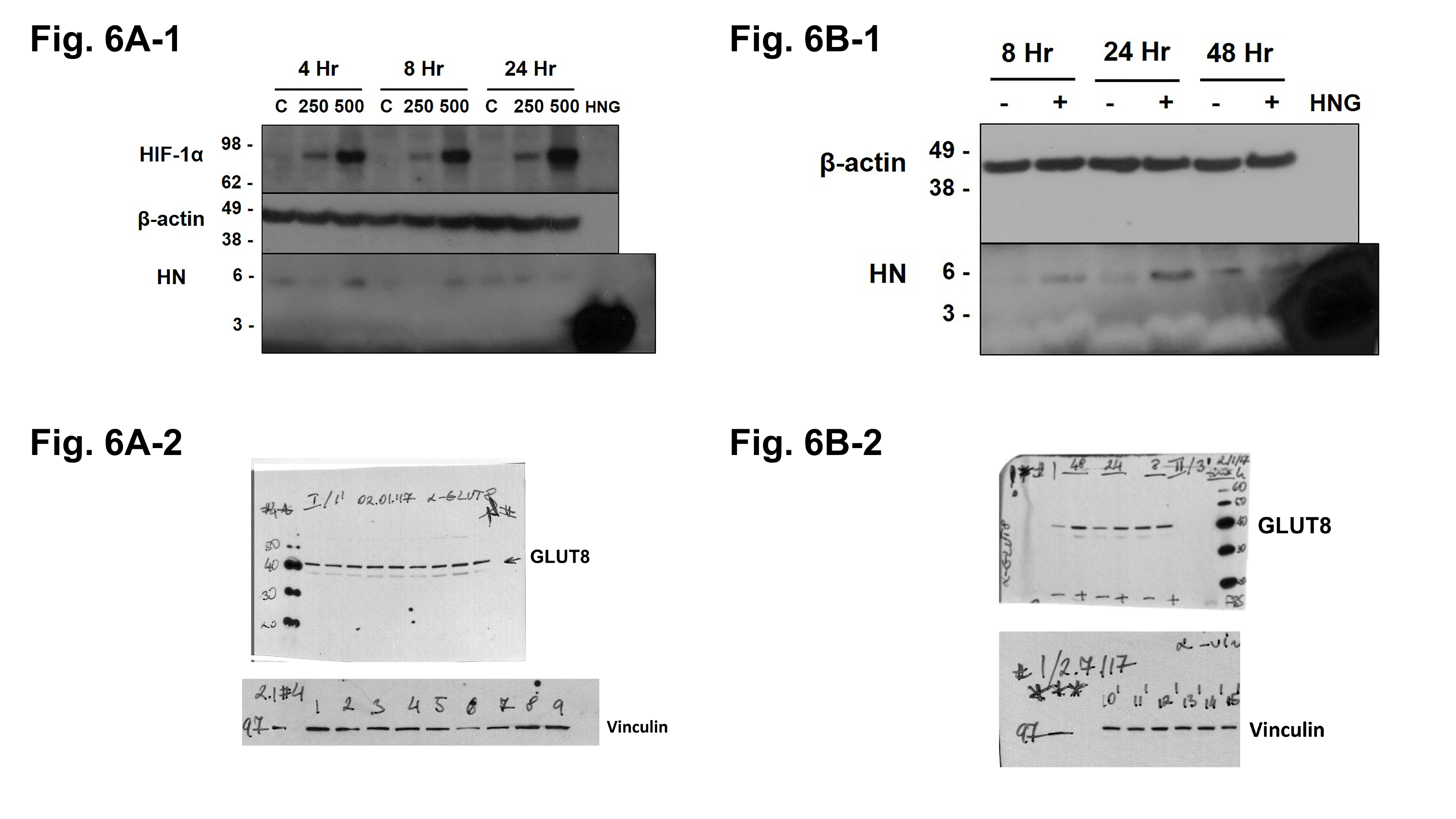

Supplement: S1 Fig — Original uncropped and unadjusted western blots used to create Fig 6 in manuscript are provided here as labeled. (TIF) [file pone.0193583.s001.tif]
